# Special endurance coefficients enable the evaluation of running performance

**DOI:** 10.1038/s41598-025-06009-6

**Published:** 2025-06-20

**Authors:** Wolfgang Blödorn, Frank Döring

**Affiliations:** Institute of Human Nutrition and Food Science, Department of Molecular Prevention, Christian-Albrechts-University of Kiel, Heinrich Hecht-Platz 10, 24118 Kiel, Germany

**Keywords:** Special endurance, Evaluating running performance, Male track running, Performance profile, Talent identification and development, Physiology, Cardiovascular biology

## Abstract

Running performance from sprint to long distance is largely determined by the interplay between basic speed and endurance. Existing power-law, physiological, and theoretical models describe and explain the characteristic decline in pace with increasing distance. However, normative and statistically validated measures that capture both the average and variability of pace decline across standard track distances remain incomplete. To address this gap, we analysed over 14,000 race times from competitive male runners and introduce the coefficient of special endurance (KsA), a novel metric that quantifies relative pace loss between adjacent race distances, from 100/200 to 5000/10,000 m. The KsA values obtained for seven distance pairs are nearly constant over decades in national runners, show low variability, and predict race times with less than one percent. The KsA-based reference ranges allow performance to be evaluated from the international to the regional level. This provides specific insight into runners’ strengths, weaknesses and progression for individualizing training, selecting the most promising race distance, and identifying and developing talent. Overall, we provide empirically derived KsA values that serve as statistical norms for pace loss from 100 m to 10,000 m to evaluate running performance of males. The current approach should also be applicable to women, juniors, and road runners.

## Introduction

Running is a fundamental trait in humans, selected through evolution^1^ and deeply rooted in physiology. Today, events ranging from the 100-m sprint to the marathon are among the most competitive sports in the world^2^. These performances are easy to assess and reflect the physiological limits of the human body^2^. As a results, performance data^3^ and the underlying physiological and biomechanical mechanisms^2^ of competitive running have been studied extensively. As early as 1906, Kennelly^4^ analyzed horse races and human running events from the 100 m sprint to the ultramarathon and discovered the universal power law relation whereby time is proportional to distance raised to the power of 9/8, indicating that doubling the distance increasing the time by a factor of 2.18 (e. g., 1500 m: 225 s; 3000 m: 225*2.18 = 490 s). Kennelly’s law and its variations have been largely confirmed by the analysis of world records in running, swimming, and other sports^5–8^. The first physiological explanation of the power law was provided by Nobel laureate Hill in 1925^5,9,10^. Hill attributed the negative exponential relationship between running pace and distance, known as the running curve, to the combined effects of anaerobic and aerobic energy production in muscles, a principle applicable to all animal movements^9^.

Applied exercise physiologists have shown that the energy cost of running per kilometer and per kilogram of body weight is almost constant (~ 1 kcal), that an individual’s maximal oxygen uptake (VO_2_max) is limited but trainable, and that VO_2_max correlates with running performance^11–13^. This led to the development of the Cooper´s 12-min run test to predict VO_2_max^14^. The relationship between runners’ performance and their physiological parameters, such as VO_2_max, fractional utilization of VO_2_max at different paces, and running economy, has been investigated through detailed running tests^12–19^. Such aerobic profiles have led to the development of easy-to-use nomograms^20^, tables^16,19,21,22^, and app-based calculators (e. g., Daniels´ VDOT calculator: https://vdoto2.com/calculator), which are widely used in running praxis to evaluate performances over different distances ranging from 1500 m to marathon.

Theoretical approaches to analyzing running performances are based on Newton’s second law^23,24^, energy considerations and the first law of thermodynamics^25^, as well as VO_2_max values and the fractional use of VO_2_max^26^. For the latter, specific nomograms are available from 1500 to 10,000 m^27^. These models have mainly been validated using world records and have a predictive accuracy of about 1–3%. Recently, two minimalist running models have been introduced based on relative metabolic power generation and a mathematical approach using local matrix completion^28–31^. They have been validated using both high and low levels performance data from GPS watches and the Great Britain Power-of-10 database^29,30^. Both models predict performance times at different performances levels with an accuracy of about 1%^28,29^.

The aforementioned approaches to describing (power law models), explaining (physiological models) and generalizing (theoretical models) pace loss with increasing race distance have been tested using world records, small sample sizes or heterogeneous data sets^3^. Despite these limitations, they have demonstrated basic validity over a wide range of distances, including ultramarathons and predominantly aerobic events^3^. However, for practical use in competitive and elite running, concrete normative values are needed that capture both average pace loss and its variability over commonly run, standardized race distances. To address this, we applied a statistical approach often used in exercise physiology (e. g. normative VO_2_max values) and medical research (e.g. normative blood pressure values)^32–34^. Specifically, we derived reference values for pace loss between neighboring distances (e.g. distance pairs such as 100 m/200 m or 800 m/1500 m) from 100 m to 10,000 m. These values are expressed as the coefficient of special endurance (KsA), a quantitative, dimensionless metric of pace loss between distance pairs.

Training experience suggests that although individual pace losses vary between adjacent race distances, they show consistently low variability in well-trained athletes. We also noticed anecdotally that runners from the 1980s had similarly low pace losses to today’s athletes, despite significant advances in training methods over time^35,36^. These observations led us to hypothesize that KsA values have low variance, remain relatively stable over decades, and are suitable for evaluating running performance from international to regional levels. They may also help to identify individual strengths and weaknesses of runners. Furthermore, we propose that KsA values can support practical decision making in talent identification, counselling and development by coaches, clubs and federations. Overall, we hypothesize that KsA values can serve as statistical norms for assessing runner performance in various practical applications.

## Materials and methods

Further details can be found in Supplementary Information on Materials and Methods.

### Dataset A1

The German Athletics Association (DLV) outdoor athletics rankings for men from 1980 to 2022 were used to compile dataset A1 (Tab. S01 and S06–012). It should be noted that the 3000 m times were also taken from the outdoor rankings. For the years 1980 to 2009 we used the printed versions of the annual best lists, while from 2010 to 2022 we used the electronic versions available at www.leichtathletik.de. We screened the annual top 30 lists for runners listed over two neighboring distances within a pair (100 m/200 m to 5000 m/10,000 m). The times of these runners were used to calculate the respective KsA values for each runner, for each distance pair, and for each year. The aggregated KsA values for each distance pair across all years from 1980 to 2022 were used to calculate statistical parameters (e.g. mean, SD, median, percentiles; Table 1). The data and statistics from 1980 to 2009 have been presented in detail previously^37^. For dataset A1, we screened a total of 10,080 performances resulting in 2875 distance pairs suitable for analysis. The original data of the dataset A1 are presented in the Supplementary Tables S06-S012.

**Table 1:** Descriptive statistics of the KsA values for pairs of neighboring distances, derived from annual best times of German male runners between 1980 and 2022.

Distance (m) pair		100/200	200/400	400/800	800/1500	1500/3000	3000/5000	5000/10,000
n		674	229	57	377	404	627	507
KsA	Mean	0.993263	0.911876	0.883943	0.919616	0.922423	0.966487	0.952898
	SD	0.013767	0.014621	0.018711	0.015990	0.019477	0.014914	0.016862
	CV (%)	1.39	1.60	2.12	1.74	2.11	1.54	1.77
	95% LCL	0.992221	0.909972	0.878978	0.917997	0.920518	0.965318	0.951426
	95% UCI	0.994304	0.913780	0.888908	0.921236	0.924328	0.967657	0.954369
	Minimum	0.954396	0.852334	0.841154	0.866677	0.785414	0.881723	0.901739
	P25	0.983886	0.904724	0.873640	0.911015	0.910779	0.957573	0.943031
	Median	0.992446	0.912000	0.887940	0.921097	0.923294	0.967013	0.953731
	P75	1.002153	0.922657	0.897323	0.929758	0.934326	0.975665	0.964642
	P90	1.011601	0.927855	0.904845	0.937127	0.944437	0.984549	0.973529
	Maximum	1.038183	0.939987	0.916627	0.970412	0.985572	1.016955	1.001483

*SD* standard deviation, *CV* Coefficient of variation, *LCL* lower confidence limit, *UCL* upper confidence limit, *P25/75/90* 25th/75th/90th percentile. underlying dataset/original data: A1 (Tab. S01).

### Datasets B1 to B6 and C1–C6

The open source performance statistics website of World Athletics (www.worldathletics.org) was used to compile the first 300 male all-time outdoor top lists for the eight distances from 100 m to 10,000 m from the World, Europe and Great Britain. The male all-time outdoor top lists over the eight distances from Germany, including West and East, and from two German states, Baden-Württemberg and Schleswig–Holstein, were retrieved from the following websites: www.leichtathletik-dgld.de, www.blv-online.de and www.shlv.de. The lists were screened for runners who were present in the respective top lists over two (Tab. S01, S013–S18) or three (Tab. S02, S19–S24) distances within a pair (100 m/200 m to 5000 m/10,000 m) or triplet (100 m/400 m, 800 m/3000 m, 1500 m/5000 m, 3000 m/10,000 m). The times for each distance pair for each runner were used to calculate the respective KsA values and derived statistical parameters (e.g. mean, SD). The KsA values and statistical parameters for the non-neighboring distances (Tab. S04) were calculated using the times of the runners who were in the top lists for the respective distance triplets. For datasets B1–B6 (Tab. S01) and C1–-C6 (Tab. S02), we screened a total of 12,593 performances resulting in 4158 distance pairs and 1121 distance triplets suitable for analysis. The original data of the datasets B1 to B6 and C1–C6 are presented in the Supplementary Tables S13–S18 (distance pairs) and S19–S24 (distance triplets), respectively. The data used for Fig. 3 are presented in the Supplementary Table S25.

### Analysis of regional middle-distance runners

The male all-time outdoor top 100 lists for the 800 m, 1500 m, and 3000 m events from two German states, Baden-Württemberg (BLV, https://www.blv-online.de/wettkampf/top-100-ewige-bestenliste) and Schleswig–Holstein (SHLV; https://www.shlv.de/ewige-bestenliste) were retrieved from the respective websites. The pdf-lists were transferred to Excel sheets and screened for runners who appeared in the top rankings of either the 800 m and 1500 m but not the 3000 m (group A), or in the 800 m, 1500 m, and 3000 m (group B). For BLV runners, we assigned 31 runners to group A and 14 to group B. For SHLV runners, we assigned 24 runners to Group A and 28 to Group B. Data are shown in Tab. S25. Statistics (see Statistics section) revealed that runners from BLV and SHLV who also appear on the regional all-time lists for the 3000 m distance have significantly (p < 0.0001) higher KsA values (~ 0.92 vs. ~ 0.90) for the 800 m/1500 m distance pair compared to those not listed in the 3000 m rankings (Fig. 3).

### Prediction error calculation

To calculate the agreement between the predicted and actual race times, we used the international, national, and regional performances of runners listed in the respective all-time rankings for 100 m, 200 m, and 400 m or for 1500 m, 3000 m, and 5000 m (Table 2). The 100 m time was used to predict the 400 m time, the 1500 m to predict the 3000 m time, and the 3000 m to predict the 5000 m time. The prediction error was expressed as the percentage deviation between the predicted and actual race times, using the absolute values of the error, regardless of whether the prediction was overestimated or underestimated. The respective median KsA values (Table 1) were used for predictions using our KsA method. For predictions using Daniels´ VDOT method (V dot 02^22^) and Riegel´s formula^7^, the respective online calculators (https://vdoto2.com/calculator; https://www.had2know.org/sports/running-time-prediction-calculator-riegel.html) were used, rounding times up or down to the nearest whole second. We noticed that the times for 1500 m and above were slightly different (e.g., ~ 10 s over 5000 m) from the VDOT tables and the VDOT online calculator. We also noticed that the exponent in Riegel’s original publication is 1.07732, while the respective online calculator uses an exponent of 1.06. For predictions using the logarithmic power law method^3,4^, the 3000 m prediction is based on both the 1500 m and 5000 m times, while the 5000 m prediction is based on both the 1500 m and 3000 m times. The pace (sec/100 m) and the logarithm of the distance length (log m) were used for linear regression analysis in such a way that the pace of the third distance formed a straight line with the two given distances. The resulting pace for the third distance was then used to predict the corresponding race time.

**Table 2:** Accuracy of predicting male performance at the international, national, and regional levels using KsA values and other approaches.

Dataset		C1	C2	C3	C4	C5	C6	
Area/contries		World	Europe	GBR	GER	BLV^1^	SHLV^2^	
Performance level		international		national		regional		
Number of runners		9	6	24	77	21	31	
100m: mean time (s) 400m: mean time (s)		9.89	10.15	10.37	10.52	10.69	10.89	
		44.36	45.40	45.54	46.38	48.27	48.37	Mean ± SD^3^
100m → 400m	KsA	− 1.47	− 1.20	0.63	0.24	− 2.13	− 0.50	1.03 ± 0.71
Prediction error (%)	VDOT	not applicable for short distances						
	Riegel	not applicable for short distances						
	LOG	not applicable, times for two distances are necessary						
Number of runners		42	59	60	100	27	60	
1500 m: mean time (sec)		211.17	213.74	218.28	220.32	227.80	233.52	
3000 m: mean time (sec)		450.38	459.96	466.10	473.02	495.26	505.32	
5000 m: mean time (sec)		775.54	791.10	805.37	815.78	858.04	881.32	Mean ± SD
1500m → 3000m prediction error (%)	KsA^4^	1.56	0.66	1.44	0.89	− 0.37	0.10	0.84 ± 0.58
	VDOT^5^	1.47	0.88	1.27	0.84	− 0.66	− 0.06	0.86 ± 0.49
	Riegel^6^	− 2.53	− 3.04	− 2.60	− 3.18	− 4.09	− 4.02	3.24 ± 0.68
	LOG^7^	− 0.73	− 1.16	− 0.57	− 0.90	− 1.12	− 0.54	0.84 ± 0.27
3000m → 5000m prediction error (%)	KsA	0.09	0.21	− 0.25	− 0.06	− 0.52	− 1.18	0.39 ± 0.42
	VDOT	− 0.38	− 0.34	1.01	− 0.82	1.00	1.22	0.80 ± 0.36
	Riegel	− 1.62	− 0.94	− 0.46	− 0.67	− 0.52	− 0.33	0.76 ± 0.47
	LOG	− 0.66	− 0.08	− 0.73	− 0.60	− 0.91	1.09	0.68 ± 0.34

^1^BLV, Badischer Leichtathletik Verband and ^2^SHLV, Schleswig-Holsteinischer Leichtathletik Verband: Regional track and field federations of Germany; ^3^The calculation was performed using the absolute values of the error, regardless of whether the prediction was overestimated or underestimated; ^4^The median KsA values, derived from the annual best times of German male runners between 1980 and 2022, of the respective neighboring (Table 1) or non-neighboring distances were used for the calculations; ^5^*V dot 02* method; ^6^Riegel´s formula; ^7^logarithmic power law method. Calculations of the prediction error for the different methods are given in the Materials and Methods section. Underlying dataset/original data: C1–C6 (Tab. S02).

### Calculation of KsA values and derived parameters

Each pair of distances (e.g., 400 m/800 m) contains a shorter neighboring distance (in meters) and a longer neighboring distance (in meters). The coefficient of special endurance (KsA) is defined as given in formula 1. KsA values for non-neighboring distances (e.g. 100 m/400 m) are calculated in the same way. The KsA values for neighboring distances (e.g. 100 m/200 m, 200 m/400 m) have been used to derive theoretical KsA values for non-neighboring distances (100 m/400 m; formula 2), the pace loss (%) within a distance pair (formula 3) and the time ratio within a distance pair (formula 4).

**Formula 1**: KsA = pace of the shorter neighboring distance (sec/100m)/ pace of the longer neighboring distance (sec/100m); given a pair of neighboring distances (e. g. 100m/200m).

**Formula 2**: Combined KsA = KsA value of the distance pair A * KsA value of the distance pair B; given two consecutive distance pairs (e.g., 100 m/200 m and 200 m/400 m).

**Formula 3**: Loss of pace (%) = [1/KsA * 100] − 100; given a pair of neighboring distances (e. g. 100m/200m).

**Formula 4**: Ratio of time = [1 + reduction of pace/100] * [length (m) of the upper distance/ length (m) of the lower distance]; given a pair of neighboring distances (e. g. 100 m/200 m).

### Illustrative example calculations using formulas 1–4

The following examples are provided for illustrative purposes only. Empirical validation of formula 1 is presented in Fig. 2, formula 2 in Fig. 4, and formula 3 and 4 in Tab. S03.

**Formula 1:** Given a sprinter with 100 m and 200 m times of 10.5 and 21.5 s, respectively, his KsA value for the 100 m/200 m distance pair is 0.9767 (10.5/ (21.5/2).

**Formula 2:** Given a middle-distance runner with KsA values of 0.90 for 200 m/400 m and 0.90 for 400 m/800 m, his combined KsA value for 200m/800m is 0.81 (0.90 × 0.90).

**Formula 3 and 4:** Given a middle-distance runner with 800m and 1500m times of 1:44.00 min (104 s, 13 s/100 m) 3:30,00 min (210 s, 14 s/100 m). The KsA value for 800m/1500m for this runner is 0.928571 (13 s/100 divided by 14 s/100m). The reduction in pace (formula 3) is 7.6924% ([1/0.928571*100] − 100), and the ratio time (formula 4) is 2.0192 ([1 + 7.6924%/100]*1500 m/800 m). A reduction in pace of 7.6924% (= 0.076924) for this particular runner means that his pace over 800m of 13 s/100m is reduced to 14 s/100m in the 1500m. Note that the pure value of 14 is higher than the pure value of 13. A time ratio of 2.0192 means that the time over 1500m (210 s) is 2.0192 times higher than the 800m time (104 s * 2.0192 = 210 s).

### Statistics

KsA calculations and other non-statistical analyses were performed in Excel, usually with at least six decimal places. The resulting data were transferred to GraphPad Prism for statistical analysis and graph generation. A p-value of less than 0.05 was considered significant. Descriptive statistics (standard deviation, coefficient of variation, confidence limits) of the KsA values (Table 1) were calculated using standard formulae. The aggregated KsA values over 42 years (Fig. S01) for each distance pair (100 m/200 m to 5000 m/10,000 m) were tested for normal distribution using the Shapiro–Wilk test. As the tests show a significant deviation from the normal distribution for the KsA values of the distance pairs 200 m/400 m (p < 0.001), 800 m/1500 m (p < 0.01), 1500 m/3000 m (p < 0.001) and 3000 m/5000 m (p < 0.001), we used percentiles (25th, median, 75th, 90th) as ranges. Percentiles have the advantage of being robust to outliers and do not require assumptions about data distribution. The percentiles of KsA values (Table 1) were obtained using the linear interpolation method. The method calculates the position of the desired percentile between the nearest rank positions (R = p*(n + 1)/100; p = percentile, n = number of data points). If the rank R is not an integer, interpolation is performed between the nearest rank positions.

**Fig. 1:**
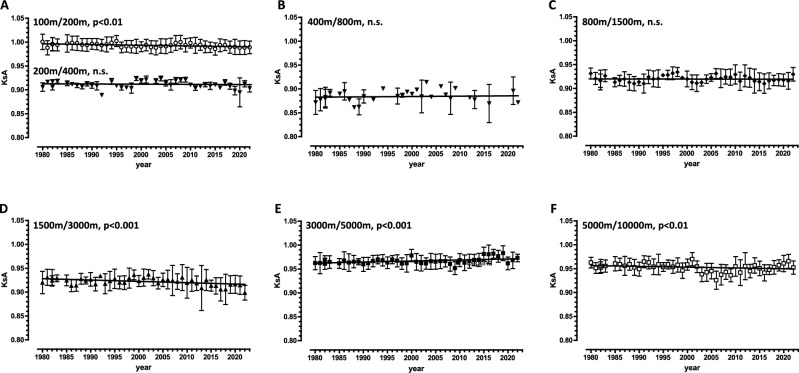
Changes in the KsA values for pairs of neighboring distances between 1980 and 2022, derived from annual best performance times of German male runners. The annual best performance times of German male runners for each year between 1980 and 2022, listed (first 30) for both neighboring distances (in meters, m) in the athletic rankings, were used to calculate the KsA values (mean ± SD) for the distance pairs 100 m/200 m and 200 m/400 m (**A**), 400 m/800 m (**B**), 800 m/1500 m (**C**), 1500 m/3000 m (**D**), 3000 m/5000 m (**E**), and 5000 m/10,000 m (**F**). Linear regression analysis was used to identify a linear trend in KsA values over the 42-year period. The p-values indicate the significance of the slope’s deviation from zero. n.s., not significant. Underlying dataset/original data: A1 (Tab. S01)/ Tab. S06–S12.

**Fig. 2:**
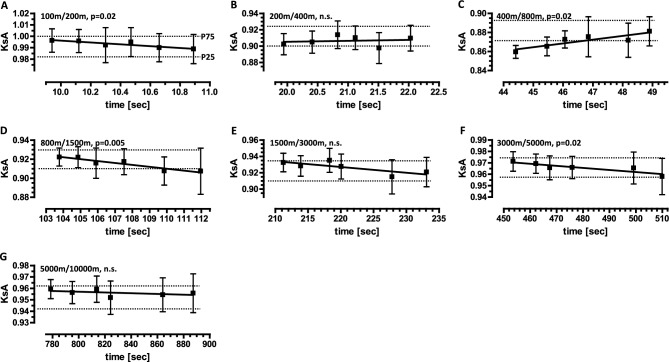
KsA values as a function of performance time at the shorter distance of a pair of neighboring distances, derived from the personal best times of male runners at the international, national, and regional levels. The personal best times of male runners at the international level (World, Europe), national level (Great Britain, Germany), and regional level (two German federal states), listed for both neighboring distances (in meters) in the athletic rankings, were used to calculate the KsA values (mean ± SD) for the distance pairs 100 m/200 m (**A**), 200 m/400 m (**B**), 400 m/800 m (**C**), 800 m/1500 m (**D**), 1500 m/3000 m (**E**), 3000 m/5000 m (**F**), and 5000 m/10,000 m (**G**). The times (mean ± SD) shown for the lower distances of each distance pair correspond, from left to right, to international, national, and regional performance categories. The upper dashed lines represent the 75th percentile (P75), and the lower dashed lines represent the 25th percentile (P25) of the KsA values, which were derived from the annual best performance times of German male runners (Table 1). Linear regression analysis was used to test whether KsA values were dependent on the performance time for the shorter distance of each distance pair. The p-values indicate the significance of the slope’s deviation from zero. n.s., not significant. Underlying datasets/original data: B1–B6 (Tab. S01)/Tab. S13–S18.

Linear regression analyses were performed to identify a linear trend in the KsA values over time (Fig. 1) or to test whether the KsA values depend on the performance time of the shorter distance of the pair of neighboring (Fig. 2) or non-neighboring (Fig. 4) distances. In this analysis, we tested whether the slope of the line was significantly different from zero. The degrees of freedom (DF) with a predictor was 1 (DFn; n = numerator). The number of DF denominators (DFd) depends on the number of data points (DFd = number of observations minus 2). The standard error of the estimate (sy.x value) was less than 0.02 in all linear regressions, indicating robust agreement between the data and the model. Non-linear regression analysis was used to identify the best-fitting curve of the trajectory of theoretical KsA values (Fig. 5). The standard errors for all parameters (e.g. K1 < 0.001) of the four resulting two-phase exponential decay functions (25^th^, median, 75^th^ and 90^th^ percentiles) are less than 0.02. The sy.x values for all four functions are less than 0.01. The half-lives of the two phases are 0.69/K1 and 0.69/K2 (K1 and K2 are given in Fig. 5B).

**Fig. 3:**
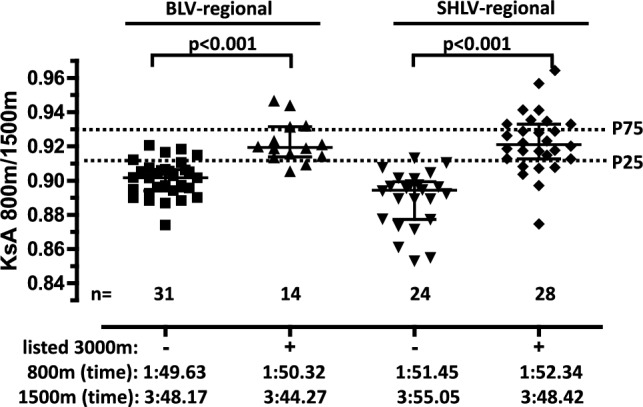
KsA values for 800 m/1500 m male regional runners, categorized by absence (−) or presence (+) in the regional all-time 3000 m rankings. Each data point represents a KsA value of an individual male runner. The values are derived from personal bests of regional runners from two German federal states (BLV, SHLV), listed for both the 800 m and the 1500 m distances. The runners were divided into four groups according to their assigned federal state and whether or not they were listed in the athletics rankings over 3000 m (listed 3000 m −/+). For each group of runners, the median and interquartile range of KsA values were plotted. The range between the median line and the lower limit represents the second quartile (25th to 50th percentile), while the range between the median line and the upper limit represents the third quartile (50th to 75th percentile). The mean times for 800 m and 1500 m are shown for each group. The lower dashed lines represent the 25th percentile (P25) and the upper dashed lines represent the 75th percentile (P75) of the KsA values derived from the annual best performance times of German male runners. The p-values indicate a significant difference between the indicated groups (two-tailed t-test for the BLV groups; Mann–Whitney U-test for the SHLV groups; Materials and Methods). BLV, Badischer Leichtathletik Verband; SHLV, Schleswig-Holsteinischer Leichtathletik Verband. Underlying datasets/original data: B5/B6/C5/C6 (Tab. S01/S02)/Tab. S25.

The regional 800 m/1500 m runners from two German states (BLV, SHLV) were each divided into two groups according to whether or not they were listed in the athletics rankings over 3000 m (Fig. 3). The KsA values of the two groups were tested separately for significant differences. The Shapiro–Wilk test showed that the KsA values of all groups were normally distributed. The test for homogeneity of variances using the Levene test showed that the KsA values of the two groups of BLV runners were homogeneous. Therefore, the KsA values of the two groups were tested for significance using a two-tailed t-test (p < 0.001). In contrast, the KsA values of the two groups of SHLV runners are not homogeneous, so the Mann–Whitney U test was used (p also < 0.001).

## Results

### The Coefficient of Special Endurance (KsA) used to analyze the performance of sprinters, middle-distance runners, and long-distance runners

We operationalize the term *special endurance*^38–40^ as the average pace, measured in seconds per 100 m (sec/100 m), achieved by an athlete in competition. We focused on the eight flat track distances ranging from 100 m to 10,000 m because these events have a notable history, are frequently run worldwide, and are represented in almost all championships. The 3000 m distance was also included because it´s also frequently run, including in junior championships. Since the loss of pace as a function of distance follows physiological principles of energy production^5^, the *Coefficient of Special Endurance (*KsA*)* is defined as the ratio between the pace at the shorter neighboring distance and the pace at the longer neighboring distance. The resulting KsA values provide a quantitative and dimensionless measure of *special endurance.* It is usually less than 1, with the exception of the KsA value for the 100 m/200 m distance pair. The closer the KsA value is to 1 for a distance pair, the better an athlete’s *special endurance* is for that particular pair, and vice versa. For example, for an athlete who runs 800 m in 1:49.23 min (13.65 s/100 m) and 1500 m in 3:42.45 min (14.83 s/100 m), the KsA value is 0.920431 (14.83 s/100 m divided by 13.65 s/100 m).

### KsA values for seven distance pairs remain remarkably constant over 42 years

To determine whether specific endurance varies over long periods of time, we examined the coefficients of specific endurance (KsA) for each year between 1980 and 2022 for German male runners who were listed in the national rankings for two neighboring distances. A total of seven distance pairs were available. It was found that the KsA values for the distance pairs 200 m/400 m (Fig. 1A), 400 m/800 m (Fig. 1B) and 800 m/1500 m (Fig. 1C) showed no dependence on the annual cycle. The KsA values for the 100 m/200 m, 1500 m/3000 m and 5000 m/10,000 m distance pairs show a decreasing trend, while the value for the 3000 m/5000 m distance pair shows an increasing trend over the 42-year period (Fig. 1A, D–F). These trends are statistically significant, but the magnitude of the changes is small (< 0.1%). The correlation coefficients are also small (r^2^ < 0.1). Thus, the KsA values obtained for German male runners have remained remarkably constant over more than four decades.

### The KsA values show a characteristic pattern of pace loss from 100 m to 10,000 m

Given the minimal variation in KsA values over a period of 42 years, we aggregated the KsA values for each distance pair over all years from 1980 to 2022. The statistics of the resulting inter- and intra-individual KsA values (Table 1, Fig. S01) show a decreasing trend in KsA values from the 100 m/200 m (median: 0.992446), to 200 m/400 m (0.912000), and to 400 m/800 m (0.887940) distance pairs. In contrast, the KsA values show an increasing trend from the 800 m/1500 m distance pair (0.921097) to 1500 m/3000 m (0.923294) and to 3000 m/5000 m (0.967013). The KsA value for the 5000 m/10,000 m distance pair (0.953731) is lower than that for the 3000 m/5000 m distance pair. The loss of pace from the shorter to the longer distance within a pair is therefore low for 3000 m/5000 m and 5000 m/10,000 m, moderate for 200 m/400 m, 800 m/1500 m and 1500 m/3000 m, and relatively high for 400 m/800 m. There is essentially no loss of pace for the 100 m/200 m distance pair. This pattern of pace loss from 100 m to 10,000 m is also present in females and in youth groups of both sexes^37^.

### The variability of the KsA values for each of the seven distance pairs is rather low

A closer look at the descriptive statistics (Table 1) of the KsA values shows that both the 95% confidence intervals and the standard deviations for each of the KsA values are very close to the mean. The coefficient of variation is less than 2.5% for all distance pairs. For further analysis, we calculated the median and the respective 25th, 75th and 90th percentiles of the KsA values for each distance pair (Table 1). The interquartile ranges differ by approximately 2.3%. For example, the KsA values for the 25th and 75th percentiles of the 800 m/1500 m distance pair are 0.911015 and 0.929758 respectively. Considering two runners, A and B, with an identical 800 m time of 1:46.00 min, the time difference between the 25th and 75th percentiles for a 1500 m run is about 5 s (runner A: 3:38.16, runner B: 3:33.77 min). This difference of around 2.3% is statistically small but relevant for running practice, indicating that runner A has potential for improvement in their 1500 m time by approximately 5 s.

### KsA values can be used as a statistical norm for the evaluation of specific endurance of runners at international, national, and regional levels

Because the KsA values obtained for the seven distance pairs have remained remarkably constant over more than four decades, show a characteristic pattern of pace loss and have low variability, we propose to adopt these KsA values as a statistical norm. This norm could be particularly useful for evaluating runners at different performance levels. To this end, we first calculated KsA values from personal best times for runners at international (World, Europe), national (Great Britain, Germany) and regional (two German states) levels. We then plotted the averaged KsA values as a function of performance time at the shorter distance within the distance pair (Fig. 2). For most distance pairs, the KsA values increase steadily with increasing performance level. These linear correlations are significant for the 100 m/200 m, 800 m/1500 m and 3000 m/5000 m distance pairs. The KsA values for the 400 m/800 m distance pair decrease significantly as the performance level improves over 400 m. Irrespective of performance level, most KsA values fall within the normative interquartile range. Taken together, the KsA values and the corresponding percentiles for neighboring distances determined from the annual best times of German runners are helpful in evaluating the performance of runners at international to regional level on the basis of personal best times. This suggests that KsA values function as an evaluation tool that is largely unaffected by the level of absolute performance.

### The normative nature of the KsA values is useful in identifying the strengths and weaknesses of runners—800 m/1500 m runners at regional level as an example

To further demonstrate the usefulness of the calculated KsA values as a statistical norm, we analyzed the performance of regional 800 m and 1500 m runners in more detail. For these middle-distance runners it is noticeable that the variance of their 800 m/1500 m KsA values is relatively high (Fig. 2D). There may be a subgroup of regional runners whose KsA values are in the range (~ 0.92) of the 75^th^ percentile, which is predominantly achieved by world class runners. Indeed, the analysis revealed that runners who are also on the regional all-time lists for the 3000 m distance have significantly (p < 0.0001) higher KsA values for the 800 m/1500 m distance pair than those who are not on the 3000 m rankings (Fig. 3; KsA values: ~ 0.92 vs. ~ 0.90). As a result, regional runners who are ranked in the 800 m, 1500 m and 3000 m events are about 6 s faster over 1500 m than those who are ranked only in the 800 m and 1500 m events, although their 800 m times are slightly slower (by ~ 0.7 s). This result highlights the importance of the upper neighboring distance (3000 m) for performance on the main distance (1500 m). Taken together, the KsA values and corresponding percentiles therefore provide an objective, quantitative and empirically based basis for identifying the strengths and weaknesses of runners’ performance.

### The normative nature of the KsA values forms the basis for establishing reference ranges for evaluating specific endurance in runners

The applicability of KsA values as a statistical norm for runners of different performance levels requires a simple tool to evaluate the specific endurance of runners. Using the 25th, median, 75th and 95th percentiles of KsA values, we defined five reference ranges from low to very high for each distance pair (Tab. S03). In addition, the parameters pace loss (%) and time ratio (dimensionless) were included in the reference ranges. These were mathematically derived from the KsA values (formulae 3 and 4). For example, an athlete who runs 800 m in 1:44.00 (104 s, 13 s/100 m) and 1500 m in 3:30.00 (210 s, 14 s/100 m) would have a KsA value of 0.9286 (13 s/100 m divided by to 14 s/100 m). This corresponds to a pace loss of 7.14% and a time ratio of 2.0192 (800 m time multiplied by 2.0192). All three endurance indices are equivalent and therefore fall into the same reference range (upper middle). They could also be useful in running practice.

### Theoretical KsA values are useful for evaluating the specific endurance of runners at international, national, and regional levels over non-neighboring distances

**Fig. 4:**
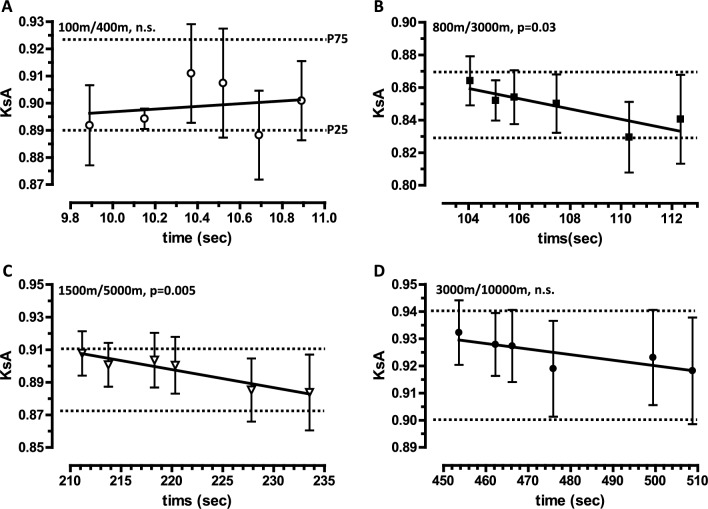
KsA values as a function of performance time at the shorter distance of a pair of non-neighboring distances, derived from personal best times of male runners at international, national and regional levels**.** Personal best times of male runners at international (World, Europe), national (Great Britain, Germany) and regional (two German states) level, listed for three distances (100 m/200 m/400 m, 800 m/1500 m/3000 m, 1500 m/3000 m/5000 m, 3000 m/5000 m/10,000 m) were used to calculate KsA values (mean ± SD) for the distance pairs 100 m/400 m (**A**), 800 m/3000 m (**B**), 1500 m/5000 m (**C**) and 3000 m/10,000 m (**D**). The times (mean ± SD) shown for the shorter distances of each distance pair correspond, from left to right, to the international, national and regional performance categories. The upper dashed lines represent the 75th percentile (P75) and the lower dashed lines represent the 25th percentile (P25) of the theoretical KsA values (Tab. S04, formula 2). These theoretical KsA values were calculated by successively multiplying the empirical single KsA values obtained from performances over neighboring distances of German male runners (Table 1). A linear regression analysis was used to test whether the KsA values were dependent on the performance time over the lower distance of each distance pair. The p-values indicate the significance of the deviation of the slope from zero. n.s., not significant. Underlying datasets/original data: C1–C6 (Tab. S02)/Tab. S19–S24.

As our empirical KsA values serve as a statistical norm for neighboring distances, we hypothesized that these values might also be useful for non-neighboring distances. We therefore used the KsA values to deduce theoretical KsA values and corresponding ranges (Tab. S04) for the distance pairs 100 m/400 m, 800 m/3000 m, 1500 m/5000 m and 3000 m/10,000 m commonly recorded by runners. To test whether the theoretical KsA values were consistent with the empirical KsA values, we analyzed the personal best times of runners at different levels. We then plotted the empirical KsA values for the non-neighboring distances against the performance times for the shorter distances within the distance pairs (Fig. 4). For all non-neighboring distance pairs and performance levels, the KsA values are within the 25th and 75th percentiles of the theoretical KsA values. The KsA values for the 800 m/3000 m and 1500 m/5000 m pairs increase steadily with higher performance level (p = 0.03 and 0.005 respectively; Fig. 4B/C). For the 100 m/400 m and 3000 m/10,000 m pairs, the KsA values show no significant correlation with performance level (Fig. 4A/D). In summary, the theoretical KsA values and corresponding ranges for the four non-neighboring distances are useful for evaluating international, national, and regional performances over non-neighboring distances.

### KsA values predict performance times with an accuracy of less than 1%

Another aspect that can be addressed using KsA values is the prediction of running times. For international, national, and regional runners, we predicted 400 m times based on 100 m times, 3000 m times based on 1500 m times and 5000 m times based on 3000 m times using our KsA approach. The prediction error obtained for the KsA method is 1.03%, 0.84% and 0.39% for the 400 m (~ 0.5 s), 3000 m (~ 3–4 s) and 5000 m (~ 3–4 s) predictions respectively (Table 2). The VDOT method, Riegel’s formula and the logarithmic power law method show similar prediction errors, but are not suitable for the 400 m prediction. In summary, our KsA approach can predict the performance of groups of runners at the international to regional level with an accuracy of less than 1%.

### The KsA values form the basis for establishing a unique function to calculate the theoretical loss of pace for each distance pair

**Fig. 5:**
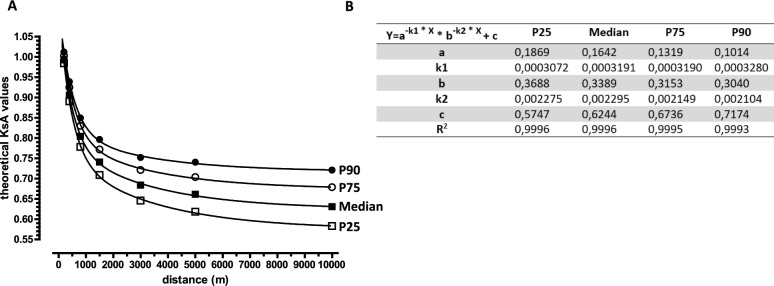
Trajectory of theoretical KsA values as a function of distance, calculated using KsA values derived from the annual best performance times of German male runners. The median and the 25th, 75th, and 90th percentiles (P25, P75, P90) of the theoretical KsA values were plotted against distance (**A**). Non-linear regression analysis was used to identify the best fitting curve of the trajectory of the theoretical KsA values. The resulting two-phase exponential decay function and its corresponding parameter are shown in the table (**B**). The *theoretical* KsA values were calculated by successively multiplying the *empirical* single KsA values obtained from performances over neighboring distances. The empirical KsA values were, in turn, derived from annual best performance times of German male runners. The course of the theoretical KsA values represents the relative loss of pace from the 100 m distance to the 10,000 m distance. Underlying dataset/original data: A1 (Tab. S01)/ Tab. S06–S12.

Due to the reliability and numerous applications of the KsA values as a statistical norm, we attempted to derive a unique function from them. To do this, we related the median and percentiles of the KsA values for the seven neighboring distance pairs in such a way that the theoretical KsA values described the loss of pace from 100 m to 10,000 m. As these pace reductions are determined by the energy supply from aerobic and anaerobic metabolic processes, we assumed a two-phase exponential decay function. In fact, such a function describes the trajectory of theoretical KsA values as a function of distance length with a correlation coefficient greater than 0.999 (Fig. 5). Using the derived functions and parameters (Fig. 5B), it is possible to calculate theoretical KsA values for any distance pair.

## Discussion

Confirming our hypotheses, we showed that KsA values 1) have remained remarkably constant over 42 years in national-level runners, 2) have low variability, 3) are suitable for evaluating runners at international, national and regional levels, 4) are useful for identifying individual strengths and weaknesses of runners (e.g. in 800 m/1500 m runners), e) can be applied to derive KsA values for non-neighboring distances, and f) allow performance prediction with an accuracy of less than 1%. To contextualize our findings, we will highlight our concept, compare it with key existing approaches, propose an evaluation framework for practical applications in running performance, and discuss the strengths and limitations of our study.

### The term *special endurance* and the *coefficient of special endurance* (KsA) – A framework for evaluating running performances

The term *endurance* has been debated in running research^3,37^. Ryder and colleagues^8^ used the term *specific endurance* for fixed-velocity running. The term *specific speed* was used for fixed-distance running, which is equivalent to running in competitions. As term *specific speed* does not capture the crucial endurance aspect of competitions, we use the term *special endurance* for this, introduced by Nabatnikowa and coworkers^38–40^. According to Nabatnikowa^41^ the level of special endurance is closely related not only to performance on the main distance, but also to performance on neighboring distances. Therefore, we operationalized the coefficient of special endurance (KsA) as the ratio between the pace at the shorter neighboring distance (sec/100 m) and the pace at the longer neighboring distance (sec/100 m) within a distance pair (e.g. 800 m/1500 m). A similar term, endurance ratio, was used to analyze 800 m and 1500 m races, but was not well defined^42^. Overall, the terms *special endurance* and *coefficient of special endurance* are conceptually sound for evaluating running performances.

### KsA values are empirically derived norms for evaluating running performance of neighboring distance pairs from 100 m to 10,000 m

We propose that the coefficient of special endurance (KsA) could serve as a statistical norm for evaluating track performances from world-class to regional levels. This applies to neighboring distances within the seven distance pairs from 100 m/200 m up to 5000 m/10,000 m.

1. The KsA values were derived from a large dataset (dataset A1) of inter- and intra-individual national performances representing an average running level between world-class and regional-class athletes.
2. We have shown that KsA values for men have remained remarkably constant over more than 40 years (1980–2022) across the seven distance pairs. This consistency is remarkable given the changes in athletes, coaches, records, equipment, and training methods over this period.
3. KsA values for female and youth categories of both sexes (U18, U20, U23) have also remained constant over 30 years, as shown previously^37^.
4. KsA values are statistically reliable and valid. This has been shown in a previous study for males, females and youth groups (U18, U20, U23) of both sexes by excluding selection bias and using the split-half method, regression analysis and variance analysis of the data^37^.
5. The variance of the KsA values is rather low, as shown by the low coefficients of variation (less than 2%) and the fact that the difference between the 25th percentile and the 75th percentile is less than 4% across all seven distance pairs.
6. The KsA values for world-class runners and regional runners differ by only about 35 percentile points.

### Empirically derived KsA values are useful for deducing theoretical KsA values for non-neighboring distance pairs

We used the *empirically* obtained KsA values for neighboring distances to calculate *theoretical* KsA values for the frequently run non-neighboring distances of 100 m/400 m, 800 m/3000 m, 1500 m/5000 m and 3000 m/10,000 m. We have shown that these theoretical KsA values are suitable for evaluating performance from world class to regional level (Fig. 4). The empirically obtained KsA values were also used to derive a mathematical function (Fig. 5) for calculating the theoretical pace loss for any distance pair. This could be useful in evaluating the performance of subsidiary distances. For example, the theoretical KsA value for the 400 m/600 m pair is 0.9342 (median; percentiles can also be calculated), indicating a pronounced pace loss between these close distances. However, we do not claim that the theoretical KsA values serve as a statistical norm. This should be achieved through statistical analysis of performance over non-neighboring and subsidiary distances.

### KsA values allow for a more nuanced evaluation of running performances than best-practice approaches

The evaluation of running performance with explicit consideration of distance pairs, such as our approach, is widely used by athletes and coaches by applying rules of thumb (e.g. 2 × 800 m time = maximum 1500 m time^43^). These rules were formulated by Nett for distances from 100 m to 10,000 m, by Coe from 400 m to marathon, by Davies-Thompson from 1500 m to 160 km, by Osler from 1 mile to marathon, and by Mercier-Leger-Desjardins from 3000 m to marathon^16,19,20,43,44^. Probabilistic models are frequently used to evaluate performances across various distances and underpin the IAAF rating scores from 100 m to marathon^19,45,46^. Application of these best practice approaches to the 400 m/800 m, 1500 m/5000 m and 5000 m/10,000 m distance pairs shows that the calculated times fall into different KsA reference ranges, ranging from low to very high (Tab. S05). We conclude that empirically derived KsA values and corresponding references ranges provide a more realistic and nuanced evaluation of runners’ performance than the relatively rigid guidelines of best practice approaches.

### KsA values can also be expressed as percentage pace loss and time ratio to accommodate different preferences in practical settings

Another approach to evaluating running performance by considering distance pairs is based on endurance indices^40,47^, which are essentially equivalent to the KsA values. These include speed reserve, endurance index and endurance coefficient (Supplementary Information on Materials and Methods; line 39ff.). Using these endurance indices, analysis of the national performances of 100 men and 100 women has shown that performance over 200 m is a key determinant of 400 m performance^47^. This result further highlights the importance of analyzing performances over neighboring distances. Because reference values for these practical indices are lacking, we have mathematically converted the KsA values into corresponding parameters (pace loss, time ratio) that are similar to the indices and may improve the applicability of the KsA approach in everyday running practice (Table S03/04). For example, a pace loss of 6.75% may be more intuitively understandable than the corresponding KsA value of 0.925.

### KsA values offer statistical norms for neighboring distances, whereas power law models describe general pace loss trends

Analysis of world records from 100 m up to ultramarathon led to the formulation of the power law with a single scaling exponent^4–8^. Verification of the power law with performance at various levels, as well as practical experience, reveals significant deviations from the law^48,49^. A detailed analysis of world records from 100 m to 10,000 m revealed a power law with two scaling exponents with a breakpoint at about 150–170 s, corresponding to a ~ 1000 m race^50^. Another study proposed a power law with six scaling exponents^51^. The limitation of a power law with one scaling exponent eventually led to the hypothesis that the relationship between pace and distance is fractal in nature^52^. In other words, the more detailed the race times and distances are analyzed, the more scaling exponents become apparent. With this in mind, we decided to directly quantify the pace loss within seven pairs of distances ranging from 100 m to 10,000 m. For this range of distances, the resulting KsA values provide quantitative norms for seven different changes in pace. A comparison of the KsA reference ranges for the distance pairs 400 m/800 m, 1500 m/5000 m and 5000 m/10,000 m with the results obtained from the power law showed no consistency (Tab. S05). Thus, the KsA values are based on specific calculations of pace loss between neighboring distances from 100 m to 10,000 m, whereas the power law models aim to capture general patterns of pace loss over a wider range of distances.

### KsA values were primarily developed to evaluate running performance, while physiology-based models serve to explain it

Analyses of running with explicit consideration of physiological principles^12–19^ have been translated into guidelines for performance over neighboring distances^15^. For example, 13:00 min for 5000 m is considered equivalent to 27:45 min for 10,000 m (Daniels VDOT calculator; https://vdoto2.com/calculator). These types of physiology-based guidelines are primarily designed for events ranging from 1500 m to the marathon^22^, 3000 m to 10,000 m^15,20^, or 5000 m to ultramarathon^16,19^. The guidelines have only been validated only in a few studies^53,54^, but they are widely used in everyday running practice, particularly Daniels’ *Running Formula*^22^. If we compare Daniels’ guidelines for three selected distance pairs with our empirically derived KsA reference ranges, we see that the 400 m/800 m time ratio is rated as very high, the 1500 m/5000 m as lower middle and the 5000 m/10,000 m as low (Tab. S05). These differences may be related to the Daniels study group and/or the primary consideration of aerobic parameters. Overall, KsA values as statistical norms may be more appropriate than physiology-based guidelines for evaluating performance from 100 m to 10,000 m, whereas physiology-based models^12–19^ are the preferred approach for explaining performance, especially over aerobic distances.

### KsA values provide statistical norms for pace loss between neighboring distances, while theoretical models offer universal formulas for pace loss

Several theoretical running models have been developed, covering distances from sprints to marathons^23–31^. These models rely on a small set of fundamental laws (e.g., Newton’s second law, the decline in pace with increasing distance) and a minimal number of empirical parameters (such as metabolic power generation and utilization). Empirical validation requires at least two race performances over different distances to enable prediction of performance at additional distances. This results in accurate modelling of performance based on individual times at two distances and the corresponding pace loss between them. Note that the pace loss between the two distances is already determined by the input times required by the models. In contrast, the KsA values, as statistical norms, are specifically designed to *evaluate* the pace loss between two distances. The theoretical models emphasize the *modelling* of performance using basic physiological and biomechanical parameters. Despite these different objectives, both approaches lead to a comparable accuracy of predicting performance of around 1%.

### Beyond performance evaluation, KsA values reflect the marked transition in energy supply between 200 and 1500 m

There has been much less research into the analysis of sprint and middle distances compared to the analysis of predominantly aerobic distances above 1500 m^2,3^. A rule of thumb states^43^ is that the optimum 200 m time should be twice the 100 m time plus 0.2 s. For the 400 m, it should be twice the 200 m time plus 3 s. The times resulting from these rules correspond to low (100 m/200 m) or high (200 m/400 m) KsA values. Analysis of middle- distance running has primarily aimed at characterizing energy supply^55–57^ and identifying parameters (e.g., maximal sprint speed) for predicting performance^3,12,28,58^. More recently, the *Running Energy Reserve Index* has been developed^59^, which predicts performance at various levels from 200 to 5000 m with an error of about 2.4%. In comparison, KsA values predict sprint and middle-distance performance with an accuracy of about 1%. Our approach provides a detailed description of the loss of pace from 200 to 1500 m. The significant loss of pace from 200 to 400 m, represented by a low KsA value, confirms data suggesting that anaerobic energy reserves—stored ATP and creatine phosphate—are sufficient up to a distance of about 240 m^60^. The considerable pace loss from 400 to 800 m and from 800 to 1500 m is probably due to the transition from predominantly anaerobic to increasingly aerobic energy production. Therefore, KsA values are not only useful for evaluating and predicting sprint and middle-distance performance, but also reflect the substantial changes in the type of energy provision from 200 to 1500m^55–57^.

### Practical application of KsA values—The pattern of four KsA values defines an evaluation scheme of running performance

**Fig. 6:**
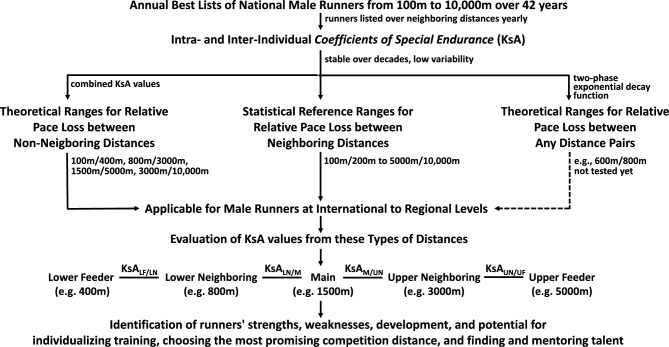
Summary of the study—approach, correlations of results, and applications for running practice. The annual best lists of national (German) male runners from 100 m to 10,000 m over 42 years (1980–2022) were used to identify the performances of these runners, which were listed annually over neighboring distances (100 m/200 m to 5000 m/10,000 m) (Table 1). The resulting intra- and inter-individual coefficients of special endurance (KsA), which represent the relative loss of pace between neighboring distances, are remarkably constant over the entire period and show low variability (Fig. 1). The median and percentiles of the KsA values (Table 1) were then used to derive respective statistical reference ranges for neighboring distances from 100 m/200 m to 5000 m/10,000 m (Tab. S03). In addition, theoretical ranges (Tab. S04) were derived for non-neighboring distances (100 m/400 m, 800 m/3000 m, 1500 m/5000 m, 3000 m/10,000 m) and a two-phase exponential decay function (Fig. 5) to calculate theoretical ranges for any distance pair. The function has not yet been tested (dashed lines). We have shown that the KsA values derived from international, national, and regional performance are largely within the respective ranges for both neighboring distances (Fig. 2) and non-neighboring distances (Fig. 3). Finally, the KsA values were used to develop an evaluation scheme. This scheme assumes that the performance of the main distance is influenced by both the performance of the respective neighboring and feeder distances (both upper and lower). From this scheme, a pattern of four different KsA values has emerged, which can be utilized for individualizing training, choosing the most promising competition distance, and finding and mentoring talents. KsA_LF/LN_, Ksa values between the lower feeder (LF) and the lower neighboring (LN) distance; KsA_LN/M_, Ksa values between the lower neighboring (LN) and the main (M) distance; KsA_M/UN_, Ksa values between the main (M) and the upper neighboring (UN) distance; KsA_UN/UF_, Ksa values between the upper neighboring (UN) and the upper feeder (UF) distance.

The KsA values for the seven pairs of neighboring distances form a chain of pace reductions from 100 m to 10,000 m, providing reference ranges for these reductions. This can be used to evaluate a runner’s performance over their main competition distance in relation to both the performance of the lower neighboring distance (lower first order neighboring distance) and the performance of the upper neighboring distance (upper first order neighboring distance). In a second step, the performances of the first-order neighboring distances are evaluated in relation to the respective performances of the second-order distances, also known as the lower and upper feeder performances. Thus, the proposed evaluation scheme consists of four KsA values (Fig. 6).

We assume that performance on the first-order neighboring distances has the greatest impact on performance on the main competition distance, and thus forms the requirement profile of a runner. Together with feeder performances, this forms the overall performance profile of a runner, which is crucial for their development. We further assume that performance on the lower neighboring distances is particularly important as it determines the maximum possible performance on the main distance. This influence is then either enhanced or diminished by the performances on the upper neighboring distances. In other words, the performance on the lower distances is the “ticket” for a particular race, while the performance on the upper distances reveals the “prospects” that the ‘ticket’ holds.

The applicability of our KsA-based approach for identifying individual strengths and weaknesses in runners was demonstrated using data from regional 800 m/1500 m athletes obtained from two German states (Fig. 3, Table S25, Material and Methods). Athletes from each state were divided into two groups based on whether or not they appeared in the 3000 m rankings (see Material and Methods): Group A runners from one German state (SHLV) had KsA values of 0.8891 ± 0.01 or (mean ± SD) and recorded times of 1:51.4 ± 2.0 min for 800 m and 3:55.0 ± 3.7 min for 1500 m. Group B had KsA values of 0.9224 ± 0.02 and ran 1:52.3 ± 1.7 min (800 m) and 3:48.4 ± 4.4 min (1500 m). Notably, a similar pattern was observed for runners from another German state (BLV). Our KsA-based interpretation of these findings is as follows: Assuming that the 1500 m is the athletes’ primary event, both groups exhibit similar performance over the lower neighboring distance (800 m), suggesting comparable performance *potential* over 1500 m. However, the higher 1500 m performance level of Group B runner (6.6 s faster) is likely attributable to their performance level of the upper neighboring distance (3000 m: 501.6 ± 14.23; KsA 1500 m/ 3000 m: 0.9224 ± 0.02). Conversely, Group A runners appear to underperform in the 1500 m, which suggest a less well-developed endurance capacity (VO_2_max) at the upper neighboring distance. Thus, we hypothesized that the upper neighboring distance (3000 m) may act as a limiting factor for Group A and as a performance-enhancing factor for Group B with respect to the main distance (1500 m). This example demonstrates how KsA values can uncover targeted performance deficits amenable to individualized training strategies.

### Strengths and limitations of the study

A key strength of this study is the establishment of empirically and statistically derived KsA values as reference ranges for pace loss between two neighboring distances of 100 m to 10,000 m. The study’s KsA reference ranges are applicable to a wide range of performances, from world-class to regional level, in contrast to studies that focus only on world-class performances. In addition, our study considers shorter distances up to 1500 m, whereas many other approaches predominantly analyze aerobic distances from 1500 m onwards. The provision of specific reference ranges for pace loss between neighboring distances allows for the straightforward identification of runners’ strengths, weaknesses, development, and potential. This is particularly advantageous in our approach, as we use the KsA values to develop an evaluation scheme that takes into account performance at both lower and upper neighboring distances relative to the main distance. This provides specific insights for individualizing training, selecting the most promising race distance, and identifying and developing talent.

A limitation of our study is the relatively small number of performances for the 400 m/800 m distance pair (57 over a period of 42 years). This makes the 400 m/800 m KsA value somewhat less certain than those for other distance pairs. Unfortunately, in almost every country in the world, only a few runners are ranked for both the 400 m and 800 m, especially when 400 m performances of 50 s or faster are considered. Another critical point of our study concerns the KsA values for the non-neighboring distances. We would like to emphasize that these are theoretical KsA values derived from two single KsA values. This also applies to the theoretical function that combines the single KsA values of all seven distance pairs. We assume that the theoretical KsA values become less certain as the gap between the two distances increases. Despite these uncertainties, we were able to demonstrate that the theoretical KsA values for non-neighboring distances, such as 100 m/400 m or 1500 m/5000 m, can be used to evaluate the performance of runners from the international to the regional level.

## Conclusion

This study provides empirically derived, statistically robust special endurance coefficients (KsA values) for male runners. KsA values are dimensionless, quantitative measure of pace loss between neighboring race distances from 100 m to 10,000 m. They serve as reference values for evaluating the performance profiles of international, national, and regional athletes, enabling identification of individual strengths, weaknesses, developmental, and performance potential. The applicability of our KsA-based approach was demonstrated through the analysis of KsA values from regional 800 m/1500 m runners, suggesting that the upper neighboring distance (3000 m) either enhanced or diminished performance over the 1500 m distance. In this way, performance at an athlete’s main race distance can be meaningfully contextualized through comparison with adjacent shorter and longer distances. Future research should extend this approach to female, junior, and road runners to assess its broader applicability. Moreover, the KsA concept may prove valuable in other sports disciplines such as race walking, swimming, or speed skating. Another research direction concerns the physiological basis of KsA values, which may lie in muscle physiology. Overall, KsA values represent a novel and useful tool for evaluating performance in male runners from 100 m to 10,000 m and hold promising potential for broader application to other athlete populations and sports disciplines.

## Supplementary Information

Supplementary Information 1.

Supplementary Information 2.

Supplementary Information 3.

Supplementary Information 4.

Supplementary Information 5.

Supplementary Information 6.

Supplementary Information 7.

Supplementary Information 8.

Supplementary Information 9.

Supplementary Information 10.

Supplementary Information 11.

Supplementary Information 12.

Supplementary Information 13.

Supplementary Information 14.

Supplementary Information 15.

Supplementary Information 16.

Supplementary Information 17.

Supplementary Information 18.

Supplementary Information 19.

Supplementary Information 20.

Supplementary Information 21.

Supplementary Information 22.

Supplementary Information 23.

Supplementary Information 24.

Supplementary Information 25.

Supplementary Information 26.

## Data Availability

All data generated or analysed during this study are included in this published article and its supplementary information files.
