# Supplementary material for: Special endurance coefficients enable the evaluation of running performance
Source: Sci Rep. 2025 Jun 20;15:20184. doi: 10.1038/s41598-025-06009-6 (PMC12181339; doi:10.1038/s41598-025-06009-6)
Supplement: Supplementary file 1 — Supplementary Information 1. [file 41598_2025_6009_MOESM1_ESM.docx]

**Supplement**

**Supplementary Information on Materials and Methods**

**Dataset A1**

From 1980 to 1993, the German annual rankings for the different categories (men, women, youth categories) for the distances 100m, 200m and 400m included both electronic and hand-timed results. There was no uniform cut-off date for all categories when hand times were no longer accepted. A detailed review of the relevant data for this study (men) shows that only a few hand-timed results were used across the distance pairs. For the 100m, for example, there are 31 hand-timed results in the dataset out of a total of 674 included times. The resulting deviation in the KsA value for the 100m/200m distance pair between “with conversion” and “without conversion” is less than 0.01%. We have therefore decided not to convert the original data. For distances of 800m and beyond, no conversion between hand times and electronic times was required according to standard practice.

**Datasets B1 to B3 and C1-C3**

Only electronic times were considered for distances between 100m and 400m. For distances of 800m and beyond, more than 98% of the times are measured electronically. The few hand times in the lists were also included (e.g. Filbert Bayi's 3:32.2 minutes over 1500m in 1974). For Great Britain (dataset B3) only 138 (400m), 185 (800m), 122 (3000m), 289 (5000m) and 238 (10,000m) performance times were available.

**Dataset B4 to B6 and C4-C6**

As the lists include electronic times and a relatively high number (>10%) of hand-timed results for the distances 100m, 200m and 400m, we adjusted the hand-timed results by adding 0.24 seconds for 100m and 200m and 0.14 seconds for 400m according to standard practice. For distances of 800m and beyond, both hand and electronic times were taken into account.

**Compilation of data and quality control**

Data from the printed versions of the annual lists of best times (dataset A1) for the years 1980 to 2009 were manually digitized into Excel spreadsheets. All other data were imported into Excel spreadsheets from their respective websites. Last name, first name and year of birth were used to identify runners listed in two or three distances. All lists were routinely checked by hand and obvious errors (e.g. misspelled names, implausible times) were cross-checked. For calculations, time measurements in minutes and seconds with two decimals were converted to seconds with two decimals.

**Calculation of the endurance indices speed reserve, endurance index, and endurance coefficient**

As discussed, endurance indices ^1,2^ are essentially equivalent to the KsA values. Both approaches refer to the relationship between the paces of two distances - one shorter and one longer - within a distance pair. The three endurance indices mentioned in the discussion section are defined as follows:

**Speed reserve**= pace (sec/100m) over the main distance minus seconds over 100m

**Endurance index**= pace (sec/100m) over the longer distance minus pace (sec/100m) over the shorter distance within a distance pair

**Endurance coefficient**= time (sec) on the longer distance divided by time (sec) on the shorter distance, multiplied by the ratio between the length (m) of the longer distance and the length (m) of the shorter distance

**Description of Supplementary Tables S06-S25**

**Tab. S01.** Description of the datasets derived from the athletics rankings of male runners to obtain performance data for pairs of neighboring distances

**Tab. S02.** Description of the datasets derived from the athletics rankings of male runners to obtain performance data for pairs of non-neighboring distances

**Tab. S02.** Description of the datasets derived from the athletics rankings of male runners to obtain performance data for pairs of non-neighboring distances

**Tab. S03.** Reference ranges for the KsA values and corresponding parameters for pairs of neighboring distances used to evaluate the performance of male runners

**Tab. S04.** Theoretical KsA ranges and corresponding parameters for pairs of non-neighboring distances used to evaluate the performance of male runners

**Tab. S05.** KsA rating categories for best possible (approach 1) or equivalent (approaches 2-8) performance over pairs of distances obtained from rules of thumb, performance ratings, empirical power laws, and physiology-based approaches

**Tab. S06-S12**: Annual best times of German male runners, listed over two neighboring distances within the pairs 100m/200m (**Tab. S06**), 200m/400m (**Tab. S07**), 400m/800m (**Tab. S08**), 800m/1500m (**Tab. S09**), 1500m/3000m (**Tab. S10**), 3000m/5000m (**Tab. S11**), and 5000m/10,000m (**Tab. S12**) for each year between 1980 and 2022. The performance times were taken from the respective **annual rankings**. Each table contains the runner's name, first name, runner´s age at which the performance was achieved, the performance times (sec) over two distances, the year in which performance was achieved, and the respective KsA value. The tables contain the original data used for Tab. 1/S03/S04 and Fig. 1/S01, respectively, in the main body of the paper.

**Tab. S13-S18:** Male runners' personal bests, listed over two neighboring distances within the seven pairs from 100m/200m to 5000m/10,000m. The runners' performances were taken from the **all-time rankings** up to the date given in Tab. S01 (date of data collection), from the World (**Tab. S13**), Europe (**Tab. S14**), Great Britain (**Tab. S15**), Germany including West and East (**Tab. S16**), and two German states, Baden-Württemberg (**Tab. S17**) and Schleswig-Holstein (**Tab. S18**). Each table contains the runner's surname, first name, date or year of birth, country if relevant (Tab. S13/14 only), performance times (sec) over two distances (pairs) and the corresponding KsA value. The tables contain the original data used for Fig. 2 in the main body of the paper**.**

**Tab. S19-S24:** Personal best times of male runners, listed over three distances within the triplets 100m/200m/400m, 800m/1500m/3000m, 1500m/3000m/5000m and 3000m/5000m/10,000m. The runners' performances were taken from the **all-time rankings** up to the date shown in Tab. S02 (date of data collection), from the World (**Tab. S19**), Europe (**Tab. S20**), Great Britain (**Tab. S21**), Germany including West and East (**Tab. S22**), and two German states, Baden-Württemberg (**Tab. S23**) and Schleswig-Holstein (**Tab. S24**). Each table contains the runner's surname, first name, date or year of birth, country if relevant (Tab. S18/19 only), performance times (sec) over three distances (triplets) and the corresponding KsA value for the non-neighboring distances. The tables contain the original data used for Fig. 4 in the main body of the paper**.**

**Tab. S25:** Personal best times of regional 800m/1500m runners who are either present (+3000m) or absent (no 3000m) on the regional all-time lists for the 3000m distance. Runners from two German states, Baden-Württemberg and Schleswig-Holstein, were included. Each table contains the runner's surname, first name, year of birth, performance times (in seconds) over two (800m, 1500m) or three distances (800m, 1500m, 3000m) and the respective KsA value for the 800m/1500m distance pair. The table provides the original data used for Fig. 3 in the main body of the paper**.**

**Supplementary Figure**


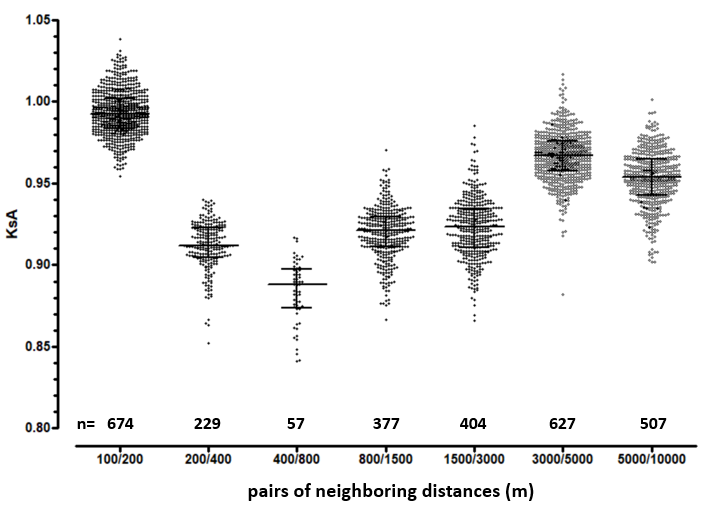


**Fig. S01. Distribution of the KsA values for pairs of neighboring distances, derived from the** **annual best performance times of German male runners between 1980 and 2022**

Each data point represents a KsA value of a single male runner for the respective distance pair (in meters, m) between 1980 and 2022. These values were derived from the annual best performance times of German runners who are listed (first 30) for both neighboring distances in the athletic rankings. The number of KsA values for each distance pair is given. For each distance pair, the median line and the interquartile range of the KsA values were plotted. The range between the median line and the lower bound represents the second quartile (25^th^ to 50^th^ percentile), while the range between the median line and the upper bound represents the third quartile (50^th^ to 75^th^ percentile).

**References**

1. Letzelter, H. Ausdauerindices und Ausdauerkoeffizienten als Gradmesser leistungs- und geschlechtsbedingter Unterschiede in der speziellen Ausdauer. *Leistungssport,* 209–217 (1981).

2. Nabatnikowa, M. J. *Die spezielle Ausdauer des Sportlers* (Bartels und Wernitz, Frankfurt am Main, 1976).
