# Supplementary material for: Special endurance coefficients enable the evaluation of running performance
Source: Sci Rep. 2025 Jun 20;15:20184. doi: 10.1038/s41598-025-06009-6 (PMC12181339; doi:10.1038/s41598-025-06009-6)
Supplement: Supplementary file 2 — Supplementary Information 2. [file 41598_2025_6009_MOESM2_ESM.docx]

**Tab. S01. Description of the datasets derived from the athletics rankings of male runners to obtain performance data for pairs of neighboring distances**

| **label** | **A1** | **B1** | **B2** | **B3** | **B4** | **B5** | **B6** |
| --- | --- | --- | --- | --- | --- | --- | --- |
| **original data** | Tab. S06-S12 | Tab. S13 | Tab. S14 | Tab. S15 | Tab. S16 | Tab. S17 | Tab. S18 |
| **data used for** | Tab. 1, S03, S04, Fig. 1/5/S01 | Fig. 2/4 | | | | | |
| **type of performances** | annual best time  for 8 distances | personal best time for 8 distances | | | | | |
| **type of ranking** | annual best lists | all-time best lists | | | | | |
| **areas/countries** | Germany | World | Europe | Great Britian | Germany | BLV^1^ | SHLV^2^ |
| **level** | national | international | | national | | regional | |
| **depth of analysis** | first 30 of every distance and year | first 300 of every distance | | first 122-300 of every distance | first 308-888 of every distance | first 100-109 of every distance | first 110-156 of every distance |
| **time period** | 1980-2022 | until 07/2023 | until 07/2023 | until 7/2023 | until 12/2022 | until 12/2021 | until 06/2023 |
| **100m/200m^3^**  **mean, n** | 10.44/21.02  674 | 9.94/19.95  124 | 10.12/20.33  139 | 10.30/20.76  183 | 10.47/21.05  447 | 10.66/21.44  67 | 10.89/22.04  85 |
| **200m/400m**  **mean, n** | 21.12/46.32  229 | 19.94/44.21  29 | 20.41/45.11  41 | 20.83/45.60  64 | 21.12/46.42  186 | 21.51/48.14  35 | 22.03/48.44  45 |
| **400m/800m**  **mean, n** | 47.10/1:46.59  57 | 44.41/1:43.34  4 | 45.46/1:45.06  6 | 46.06/1:45.56  9 | 46.85/1:47.07  52 | 48.19/1:49.53  15 | 48.90/1:51.01  28 |
| **800m/1500m**  **mean, n** | 1:48.47/3:41.21  377 | 1:43.77/3:30.98  53 | 1:44.86-3:33.25  71 | 1:45.90/3:36.87  70 | 1:47.52/3:39.79  162 | 1:49.84/3:46.96  45 | 1:51.96/3:51.46  54 |
| **1500m/3000m**  **mean, n** | 3:42.99/8:03.67  404 | 3:31.33/7:33.29  81 | 3:33.88/7:40.86  93 | 3:38.28/7:46.90  73 | 3:40.05/7:54.54  137 | 3:47.80/8:18.12  42 | 3:53.08/8:26.40  75 |
| **3000m/5000m**  **mean, n** | 8:05.15/13:56.72  627 | 7:33.52/12:58.31  185 | 7:42.17/13:14.76  199 | 7:47.45/13:26.90  98 | 7:55.89/13:41.22  198 | 8:19.13/14:21.74  64 | 8:29.93/14:47.37  95 |
| **5000m/10,000m**  **mean, n** | 14:00.29/29:24.15  507 | 12:58.70/27:03.42  144 | 13:15.13/27:42.95  163 | 13:33.75/28:16.72  150 | 13:44.31/28:52.34  213 | 14:24.08/30:11.11  57 | 14:47.31/30:57.04  77 |

^1^BLV, Badischer Leichtathletik Verband and ^2^SHLV, Schleswig-Holsteinischer Leichtathletik Verband: Regional track and field federations of Germany;^3^times are given in seconds (100m-400m) or minutes (800m-10,000m) with two decimal places.
