# Supplementary material for: Special endurance coefficients enable the evaluation of running performance
Source: Sci Rep. 2025 Jun 20;15:20184. doi: 10.1038/s41598-025-06009-6 (PMC12181339; doi:10.1038/s41598-025-06009-6)
Supplement: Supplementary file 3 — Supplementary Information 3. [file 41598_2025_6009_MOESM3_ESM.docx]

**Tab. S02. Description of the datasets derived from the athletics rankings of male runners to obtain performance data for pairs of non-neighboring distances**

| **label** | **C1** | **C2** | **C3** | **C4** | **C5** | **C6** |
| --- | --- | --- | --- | --- | --- | --- |
| **original data** | Tab. S19 | Tab. S20 | Tab. S21 | Tab. S22 | Tab. S23 | Tab. S24 |
| **data used for** | Tab. 2, Fig. 3/4 | | | | | |
| **type of performance** | personal best time for 8 distances | | | | | |
| **type of ranking** | all-time best lists | | | | | |
| **areas/countries** | World | Europe | Great Britian | Germany | BLV^1^ | SHLV^2^ |
| **level** | international | | national | | regional | |
| **depth of analysis** | first 300 of every distance | | first 122-300 of every distance | first 308-888 of every distance | first 100-109 of every distance | first 110-156 of every distance |
| **time period** | until 07/2023 | until 07/2023 | until 7/2023 | until 12/2022 | until 12/2021 | until 06/2023 |
| **100m/400m^3^**  **mean, n** | 9.89/44.36  9 | 10.15/45.40  6 | 10.37/45.54  24 | 10.52/46.38  77 | 10.69/48.27  21 | 10.89/48.38  31 |
| **800/3000m**  **mean, n** | 1:44.06/7:31.69  4 | 1:45.80/7:42.49  14 | 1:45.80/7:44.63  9 | 1:47.46/7:54.11  44 | 1:50.32/8:18.89  14 | 1:52.34/8:21.60  28 |
| **1500m/5000m**  **mean, n** | 3:31.17/12:55.54  42 | 3:33.74/13:11.10  59 | 3:38.28/13:25.37  60 | 3:40.32/13:35.78  100 | 3:47.80/14:18.04  27 | 3:53.52/14:41.32  60 |
| **3000m/10000m**  **mean, n** | 7:33.65/27:02.87  89 | 7:42.26/27:40.75  116 | 7:46.24/27:56.24  56 | 7:55.92/28:46.90  136 | 8:19.44/30:04.04  40 | 8:28.75/30:47.63  55 |

^1^BLV, Badischer Leichtathletik Verband and ^2^SHLV, Schleswig-Holsteinischer Leichtathletik Verband: Regional track and field federations of Germany; ^3^times are given in seconds (100m-400m) or minutes (800m-10,000m) with two decimal places.
