# Supplementary material for: Special endurance coefficients enable the evaluation of running performance
Source: Sci Rep. 2025 Jun 20;15:20184. doi: 10.1038/s41598-025-06009-6 (PMC12181339; doi:10.1038/s41598-025-06009-6)
Supplement: Supplementary file 4 — Supplementary Information 4. [file 41598_2025_6009_MOESM4_ESM.docx]

**Tab. S03.** **Reference ranges for the KsA values and corresponding parameters for pairs of neighboring distances used to evaluate the performance of male runners**

| **distance (m) pair** | **parameter** | **reference ranges** | | | | |
| --- | --- | --- | --- | --- | --- | --- |
|  |  | **very high** | **high** | **upper middle** | **lower middle** | **low** |
| **100/**  **200** | **KsA^1^** | ≥1.0116 | 1.0115-1.0022 | 1.0021-0.9924 | 0.9923-0.9840 | ≤0.9839 |
|  | **pace loss (%)^2^** | ≤-1.1468 | -1.1467- -0.2148 | -0.2147-0.7611 | 0.7612-1.6378 | ≥1.6378 |
|  | **time ratio^3^** | ≤1.9771 | 1.9772-1.9957 | 1.9958-2.0152 | 2.0153-2.0328 | ≥2.0328 |
| **200/**  **400** | **KsA** | ≥0.9279 | 0.9278-0.9227 | 0.9226-0.9120 | 0.9119-0.9048 | ≤0.9047 |
|  | **pace loss (%)** | ≤7.7755 | 7.7756-8.3826 | 8.3827-9.6491 | 9.6492-10.5309 | ≥10.5309 |
|  | **time ratio** | ≤2.1555 | 2.1556-2.1677 | 2.1678-2.1930 | 2.1931-2.2106 | ≥2.2106 |
| **400/**  **800** | **KsA** | ≥0.9049 | 0.9048-0.8973 | 0.8972-0.8879 | 0.8878-0.8737 | ≤0.8736 |
|  | **pace loss (%)** | ≤10.5162 | 10.5163-11.4426 | 11.4427-12.6202 | 12.6203-14.4636 | ≥14.4636 |
|  | **time ratio** | ≤2.2103 | 2.2104-2.2289 | 2.2290-2.2524 | 2.2525-2.2893 | ≥2.2893 |
| **800/**  **1500** | **KsA** | ≥0.9371 | 0.9370-0.9298 | 0.9297-0.9211 | 0.9210-0.9111 | ≤0.9110 |
|  | **pace loss (%)** | ≤6.7091 | 6.7092-7.5549 | 7.5550-8.5662 | 8.5663-9.7677 | ≥9.7677 |
|  | **time ratio** | ≤2.0008 | 2.0010-2.0167 | 2.0168-2.0356 | 2.0357-2.0581 | ≥2.0581 |
| **1500/**  **3000** | **KsA** | ≥0.9444 | 0.9443-0.9343 | 0.9342-0.9233 | 0.9232-0.9109 | ≤0.9108 |
|  | **pace loss (%)** | ≤5.8832 | 5.8833-7.0290 | 7.0291-8.3079 | 8.3080-9.7961 | ≥9.7961 |
|  | **time ratio** | ≤2.1177 | 2.1178-2.1406 | 2.1407-2.1662 | 2.1663-2.1959 | ≥2.1959 |
| **3000/**  **5000** | **KsA** | ≥0.9846 | 0.9845-0.9757 | 0.9756-0.9670 | 0.9669-0.9577 | ≤0.9576 |
|  | **pace loss (%)** | ≤1.5693 | 1.5694-2.4942 | 2.4943-3.4112 | 3.4113-4.4307 | ≥4.4307 |
|  | **time ratio** | ≤1.6928 | 1.6929-1.7082 | 1.7083-1.7235 | 1.7236-1.7405 | ≥1.7405 |
| **5000/**  **10,000** | **KsA** | ≥0.9735 | 0.9734-0.9646 | 0.9645-0.9537 | 0.9536-0.9431 | ≤0.9430 |
|  | **pace loss (%)** | ≤2.7191 | 2.7192-3.6654 | 3.6655-4.8514 | 4.8515-6.0411 | ≥6.0411 |
|  | **time ratio** | ≤2.0544 | 2.0545-2.0733 | 2.0734-2.0970 | 2.0971-2.1208 | ≥2.1208 |

^1^The reference ranges refer to the respective percentiles of the KsA values (Tab. 1) which were derived from the annual best times of German male runners from 1980 to 2022; ^2^pace loss (%) and ^3^time ratio (dimensionless) were mathematically derived (formula 3 and 4, Materials and Methods) from the respective KsA values. Note, that the values are calculated with 6 decimal places. Underlying dataset/original data: A1 (Tab. S01)/ Tab. S06-S12.
