# Supplementary material for: Special endurance coefficients enable the evaluation of running performance
Source: Sci Rep. 2025 Jun 20;15:20184. doi: 10.1038/s41598-025-06009-6 (PMC12181339; doi:10.1038/s41598-025-06009-6)
Supplement: Supplementary file 5 — Supplementary Information 5. [file 41598_2025_6009_MOESM5_ESM.docx]

**Tab. S04. Theoretical KsA ranges and corresponding parameters for pairs of non-neighboring distances used to evaluate the performance of male runners**

| **distance (m) pair** | **parameter** | **theoretical ranges** | | | | |
| --- | --- | --- | --- | --- | --- | --- |
|  |  | **very high** | **high** | **upper middle** | **lower middle** | **low** |
| **100/400** | **theoretical KsA^1^** | ≥0.9386 | 0.9385-0.9245 | 0.9244-0.9052 | 0.9051-0.8902 | ≤0.8901 |
|  | **pase loss (%)^2^** | ≤6.5395 | 6.5396-8.1498 | 8.1499-10.4837 | 10.4838-12.3411 | ≥12.3412 |
|  | **time ratio^3^** | ≤ 4.2616 | 4.2617-4.3260 | 4.3261-4.4193 | 4.4194-4.4935 | ≥4.4936 |
| **800/3000** | **theoretical KsA** | ≥0.8851 | 0.8850-0.8688 | 0.8687-0.8505 | 0.8504-0.8298 | ≤0.8297 |
|  | **pace loss (%)** | ≤12.9870 | 12.9871-15.1149 | 15.1150-17.5857 | 17.5858-20.5205 | ≥20.5206 |
|  | **time ratio** | ≤4.2370 | 4.2371-4.3168 | 4.3169-4.4095 | 4.4096-4.5194 | ≥4.5195 |
| **1500/5000** | **theoretical KsA** | ≥0.9298 | 0.9297-0.9115 | 0.9114-0.8929 | 0.8928-0.8722 | ≤0.8721 |
|  | **pace loss (%)** | ≤7.5449 | 7.5450-9.6985 | 9.6986-12.0025 | 12.0026-14.6607 | ≥14.6608 |
|  | **time ratio** | ≤3.5848 | 3.5849-3.6566 | 3.6567-3.7334 | 3.7335-3.8219 | ≥3.8220 |
| **3000/10,000** | **theoretical KsA** | ≥0.9585 | 0.9584-0.9413 | 0.9412-0.9224 | 0.9223-0.9031 | ≤0.9030 |
|  | **pace loss (%)** | ≤4.3311 | 4.3312-6.2510 | 6.2511-8.4281 | 8.4282-10.7393 | ≥10.7394 |
|  | **time ratio** | ≤3.4777 | 3.4778-3.5417 | 3.5418-3.6142 | 3.6143-3.6912 | ≥3.6913 |

^1^The theoretical ranges for the KsA values of non-neighboring distances were obtained (formula 2, Materials and Methods) from the respective percentiles of the single KsA values of neighboring distances (Tab. 1). In turn, the single KsA values were derived from annual best times of German male runners from 1980 to 2022; ^2^pace loss (%) and ^3^time ratio were mathematically derived (formula 3 and 4, Materials and Methods) from the respective theoretical KsA values. Note, that the values are calculated with 6 decimal places. Underlying dataset/original data: A1 (Tab. S01)/ Tab. S06-S12.
