# Supplementary material for: Special endurance coefficients enable the evaluation of running performance
Source: Sci Rep. 2025 Jun 20;15:20184. doi: 10.1038/s41598-025-06009-6 (PMC12181339; doi:10.1038/s41598-025-06009-6)
Supplement: Supplementary file 6 — Supplementary Information 6. [file 41598_2025_6009_MOESM6_ESM.docx]

**Tab. S05. KsA rating categories for best possible (approach 1) or equivalent (approaches 2-8) performance over pairs of distances obtained from rules of thumb, performance ratings, empirical power laws, and physiology-based approaches**

| **author** | **ref.** | **approach** | **calculation**  **(times in sec)** | **400m (sec)** | **800m (sec)** | **KsA values** | **KsA reference range** |
| --- | --- | --- | --- | --- | --- | --- | --- |
| Nett^1^ | 84 | rule of thumb | 400m time+60 | 47.00 | 107.00 | 0.8785 | **lower middle** |
| Coe^2^ | 76 | rule of thumb | 400m time*2.1818 | 47.00 | 102.54 | 0.9167 | **very high** |
| IAAF scores^3^ | na | performance rating | scores | 47.00 | 109.33 | 0.8598 | **low** |
| Osler^4^ | 85 | performance rating | na | na | na | na | na |
| Kennelly^5^ | 4 | empirically, power law | 400m time*2.1799 | 47.00 | 102.46 | 0.9175 | **very high** |
| Riegel^6^ | 8 | empirically, power law | calculator | 47.00 | 97.00 | 0.9691 | **very high** |
| Davies/Thompson^7^ | 44, 85 | physiology | na | na | na | na | na |
| Daniels^8^ | 72 | physiology | table 6.1 (ref. 72) | 47.00 | 103.40 | 0.9091 | **very high** |
| **author** | **ref.** | **approach** | **calculation**  **(times in sec)** | **1500m (sec)** | **5000m (sec)** | **KsA values** | **KsA reference range** |
| Nett | 84 | rule of thumb | ((2x1500 time +25)+20)/3*5 | 212.00 | 781.67 | 0.9138 | **high** |
| Coe | 76 | rule of thumb | 1500m time/0.27 | 212.00 | 785.19 | 0.9000 | **upper middle** |
| IAAF scores | na | performance rating | equivalent scores | 212.00 | 778.20 | 0.9081 | **upper middle** |
| Osler | 85 | performance rating | na | na | na | na | na |
| Kennelly | 4 | empirically, power law | 1500m time*3.8757 | 212.00 | 821.65 | 0.8601 | **low** |
| Riegel | 8 | empirically, power law | calculator | 212.00 | 759.00 | 0.9310 | **very high** |
| Davies/Thompson | 44, 85 | physiology | na | na | na | na | na |
| Daniels | 72 | physiology | VDOT calculator | 212.00 | 800.00 | 0.8833 | **lower middle** |
| **author** | **ref.** | **approach** | **calculation**  **(times in sec)** | **5000m (sec)** | **10,000m (sec)** | **KsA values** | **KsA reference range** |
| Nett | 84 | rule of thumb | 2x5000m time+60 | 780.00 | 1620.00 | 0.9630 | **upper middle** |
| Coe | 76 | rule of thumb | 5000 time*2.0833 | 780.00 | 1624.97 | 0.9600 | **upper middle** |
| IAAF scores | na | performance rating | equivalent scores | 780.00 | 1630.00 | 0.9571 | **upper middle** |
| Osler | 85 | performance rating | table 2.6 | 780.00 | 1715.00 | 0.9096 | **low** |
| Kennelly | 4 | empirically, power law | 5000m time*2.181 | 780.00 | 1701.18 | 0.9170 | **low** |
| Riegel | 8 | empirically, power law | calulator | 780.00 | 1626.00 | 0.9594 | **upper middle** |
| Davies/Thompson | 44, 85 | physiology | table 2.3 (ref. 85) | 780.00 | 1746.00 | 0.8935 | **low** |
| Daniels | 72 | physiology | VDOT calculator | 780.00 | 1665.00 | 0.9369 | **low** |

Endurance indices ^1,2^ which are essentially equivalent to the KsA values, were not considered in this listing.

^1^Nett's ^3^ rules provide the best possible time for the longer distance when the time for the shorter distance is given within two neighboring distances. For the 400m/800m distance pair, Nett provides different rules depending on the 400m time. The rule given here applies to 400m times between 46 and 51 seconds. For the 1500m/5000m distance pair, the rules for the 1500m/3000m (2x1500m time+25 sec) and 3000m/5000m ((3000m time+20 sec)/3*5) distance pairs have been combined.

^2^Coe´s ^4^ rules distinguish between middle-distance runners (400m/800m pair) and track long-distance runners (1500m/5000m, 5000m/10,000m). The calculated times were taken from the relevant tables.

^3^The IAAF (International Association of Athletics Federations) scoring points were determined using the respective online-calculator (https://caltaf.com/pointscalc/calc.html).

^4^Osler´s ^5^ equivalent running times from 1 mile to marathon are based on a performance rating, similar to the IAAF scoring point. The calculated times were taken from the corresponding table.

^5^Calculations were made using Kennelly´s power law ^6^: time is proportional to distance^9/8^.

^6^Calculations were made using an online calculator (https://www.had2know.org/sports/ running-time-prediction-calculator-riegel.html) based on Riegel´s power law: time of longer distance= time of shorter distance*(length of longer distance/length of shorter distance)^1.06^. We noted that the exponent in Riegel's original publication is 1.07732 ^7^.

^7^Davies and Thompson's ^5,8^ equivalent running times from 1500m to ultramarathon are based on VO_2_max, fractional utilization of VO_2_max, and an assumed running economy. The times (5000m/10,000m) were taken from the relevant table.

^8^Daniels ^9^ equivalent running times from 1500 to marathon are based on VO_2_max, fractional utilization of VO_2_max, average running economy, and targeted running tests. The times were taken from the relevant tables (400m/800m) or computed (1500m/5000m, 5000m/10,000m) using the VDOT online calculator (https://vdoto2.com/calculator). We noted that the times for 1500m and above differ slightly (e.g., ~10 seconds over 5000m) between the tables and the online calculator.

na, not applicable; ref, reference; KsA reference ranges refer to the information in Tab. S03 and S04.

**References**

1. Letzelter, H. Ausdauerindices und Ausdauerkoeffizienten als Gradmesser leistungs- und geschlechtsbedingter Unterschiede in der speziellen Ausdauer. *Leistungssport,* 209–217 (1981).

2. Nabatnikowa, M. J. *Die spezielle Ausdauer des Sportlers* (Bartels und Wernitz, Frankfurt am Main, 1976).

3. Nett, T. *Training des Kurz-, Mittel- und Langstreckenläufers.* 3rd ed. (Wilhelm Limpert Verlag, Frankfurt am Main, 1956).

4. Martin, D., E. & Coe, P., N. *Training distance runners* (Leisure Press, 1991).

5. Noakes, T. *Lore of running.* 4th ed. (Human Kinetics, USA, 2003).

6. Kennelly, A. E. An Approximate Law of Fatigue in the Speeds of Racing Animals. *Proceedings of the American Academy of Arts and Sciences,* 275–331 (1906).

7. Riegel, P. S. Athletic records and human endurance. *American scientist* **69,** 285–290 (1981).

8. Davies, C. T. & Thompson, M. W. Aerobic performance of female marathon and male ultramarathon athletes. *European journal of applied physiology and occupational physiology* **41,** 233–245; 10.1007/BF00429740 (1979).

9. Daniels, J. *Daniels´ running formula* (Human Kinetics, USA, 2014).
